# Navigating the Landscape of Periodontitis Nonsurgical Treatment: A Metatrend Study of The Scientific Production and Trends From 2001-2020

**DOI:** 10.1590/0103-6440202406110

**Published:** 2024-10-25

**Authors:** Victoria L Abdo, Caroline Dini, Maria Helena R Borges, Danilo V A P Domingues, Kamily A C C Sanchez, Rodrigo Martins, Belen Retamal-Valdes, Valentim A R Barão, Joāo Gabriel S Souza

**Affiliations:** 1 Department of Periodontology, Dental Research Division, Guarulhos University, Praça Tereza Cristina, 88 - Centro, Guarulhos, São Paulo 07023-070, Brazil; 2 Department of Prosthodontics and Periodontology, Piracicaba Dental SchoolUniversidade Estadual de Campinas (UNICAMP). Av. Limeira, 901, Piracicaba, São Paulo 13414-903, Brazil.

**Keywords:** bibliometric analysis, non-surgical treatment, periodontitis

## Abstract

Nonsurgical therapies have been recommended and employed as a less invasive and cost-effective modality in managing periodontitis. In this context, different therapeutic protocols have been tested in the last decades. Therefore, mapping the scientific trends and patterns provides critical insights into the state of research in the field, which has not been explored for overall nonsurgical periodontitis treatment studies. Articles from 2001 to 2020 were retrieved from the Web of Science database using appropriate terms and keywords. Article selection and data extraction were performed by calibrated examiners. All articles focusing on nonsurgical periodontitis treatment were included. Descriptive, bivariate, and multivariate logistic regression analyses were conducted. 1,519 articles were included. Randomized clinical trials (RCTs) were the most used design (44.1%), and professional biofilm control was the topic most studied (35.6%). Europe published the most significant number of articles (41.1%). The USA was the country that collaborated more with other countries. Asia (p<0.001), South America (p=0.004), and Oceania/Africa (p=0.016) showed a lower chance to have international collaboration. Studies from North America were more likely to be RCTs than studies from Europe (p=0.050); studies focusing on professional biofilm control (p<0.001) and other topics (p<0.001) were less likely to be evaluated by RCTs. The nonsurgical periodontitis treatment field mainly conducted RCTs, and the topic most explored by all studies was professional biofilm control. International collaboration and conduct of RCTs in this field occurred mainly among high-income countries. Decentralizing scientific resources, making integrative connections globally, and evaluating new topics may improve evidence-based periodontology.

## Resumo

As terapias não cirúrgicas têm sido recomendadas e empregadas como uma modalidade menos invasiva e econômica no manejo da periodontite. Nesse contexto, diferentes protocolos terapêuticos têm sido testados nas últimas décadas. Portanto, mapear as tendências e padrões científicos fornece insights críticos sobre o estado da pesquisa na área, o que ainda não foi explorado em estudos gerais de tratamento não cirúrgico da periodontite. Artigos de 2001 a 2020 foram analisados na base Web of Science usando termos/palavras-chave apropriados. A seleção dos artigos e a extração dos dados foram realizadas por examinadores calibrados. Todos os artigos focados no tratamento não cirúrgico da periodontite foram incluídos. Foram realizadas análises descritivas, bivariadas e de regressão logística multivariada. Foram incluídos 1.519 artigos. Ensaios clínicos randomizados (ECR) foram o design mais utilizado (44,1%), e o controle profissional do biofilme foi o tema mais estudado (35,6%). A Europa publicou o maior número de artigos (41,1%). Os EUA foi o país que mais colaborou com outros países. Ásia (p<0,001), América do Sul (p=0,004) e Oceania/África (p=0,016) mostraram menor chance de ter colaboração internacional. Estudos da América do Norte tinham mais probabilidade de serem ECR do que estudos da Europa (p=0,050); estudos focados no controle profissional do biofilme (p<0,001) e outros tópicos (p<0,001) tinham menos probabilidade de serem avaliados por ECR. O campo de tratamento não cirúrgico da periodontite conduziu principalmente ECRs e o tema mais explorado por todos os estudos foi o controle profissional do biofilme. A colaboração internacional e a condução de ECRs neste campo ocorreram principalmente entre países de alta renda. Descentralizar os recursos científicos, estabelecer conexões integrativas globalmente e avaliar novos tópicos podem melhorar a Periodontia baseada em evidências.

**Figure:**
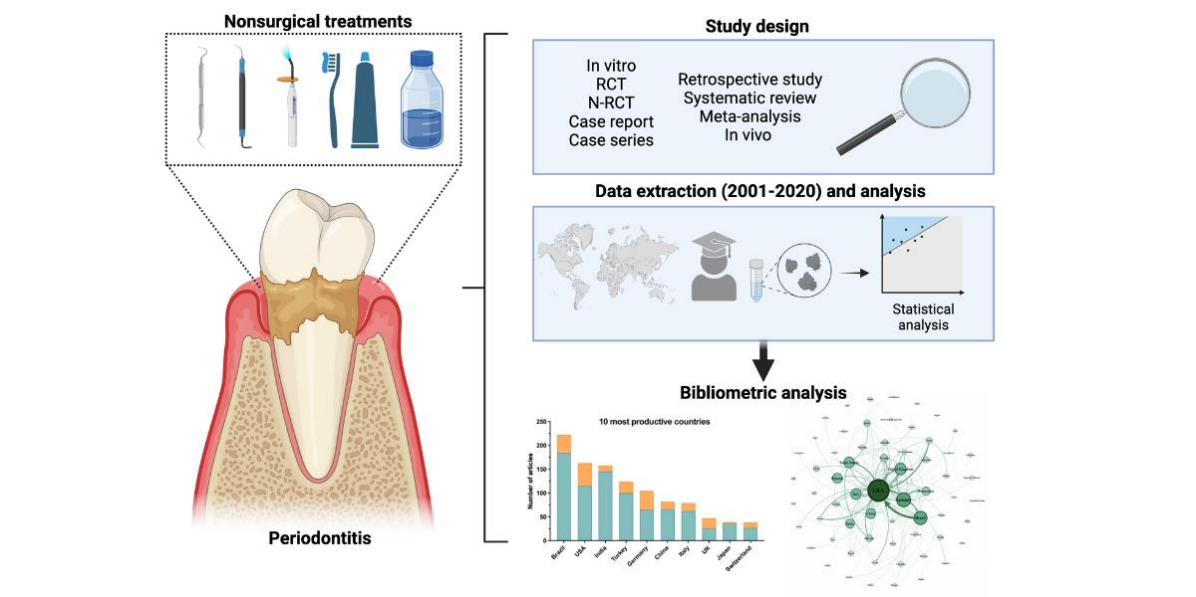

## Introduction

Periodontal diseases are chronic inflammatory and multifactorial conditions that affect tooth-supporting structures, including gingival tissues and alveolar bone, considered an epidemic and a significant global public health concern^1^. Most of these conditions are induced by polymicrobial biofilm accumulation, which leads to inflammatory responses in the host tissues^2^. Although poor oral hygiene plays a significant role in inducing periodontal diseases, different risk factors have been directly linked to triggering periodontal inflammatory responses, such as smoking, aging, and systemic conditions^3^ ^,^ ^4^ ^,^ ^5^. These conditions include a range of clinical disorders, with gingivitis and periodontitis being the most prevalent and well-studied among them^6^ ^,^ ^7^. According to the last classification consensus report, periodontitis is directly associated with and induced by dysbiotic polymicrobial biofilms and is characterized by the progressive destruction of periodontal structures^1^. Moreover, when left untreated, periodontitis can lead to tooth loss, compromising chewing function, aesthetics, and quality of life, and has been associated with a bidirectional relationship with systemic diseases such as diabetes and cardiovascular diseases^1^ ^,^ ^8^ ^,^ ^12^. In 2010, severe periodontitis globally affected approximately 740 million people, creating a substantial burden for healthcare systems and posing a public health challenge^13^. Moreover, previous evidence indicates a significant increase in periodontal disease's global burden from 1990 to 2019^14^. Considering the high prevalence of these conditions and the serious health consequences, including systemic conditions, researchers and clinicians have extensively explored effective therapeutic strategies^15^.

Although for decades the surgical treatment for periodontitis was considered the leading choice for restoring periodontal health, a paradigm shift in periodontal therapy considering nonsurgical approaches led to new perspectives to deal with the disease’s clinical signs using less invasive protocols^16^. Considering the multifactorial nature of this condition, numerous therapeutic interventions have been applied to achieve effective treatment^17^. Nonsurgical modalities comprise a broad range of interventions, including different instruments for mechanical debridement^18^. Although scaling and root planning have been considered the gold-standard nonsurgical treatment, it is often associated with adjuvants as chemical agents, host-modulating agents (local or systemic), and local and systemic antimicrobials^18^, or even followed by surgical interventions ^17^ ^,^ ^18^. Interestingly, there is no treatment recipe for all patients since patients and biological patterns affect disease progression and treatment responses^19^. Moreover, oral hygiene clearly improves clinical signs of the disease and must be considered for all modalities^17^. The main goal of periodontal therapy is to recover microbial symbiosis and, consequently, periodontal tissue health^20^. In this context, different instruments, techniques, and agents have been developed and tested in the last decades to enhance biofilm removal and host responses during periodontal therapy^21^. Therefore, mapping the scientific trends and patterns regarding periodontal nonsurgical treatments is a powerful tool to systematically analyze and concisely summarize important aspects of this topic and provide critical insights into the state of research in the field^22^.

Bibliometric studies have explored the scientific trends and quality in the studies evaluating the relationship between periodontal therapy and cardiovascular disease^23^ regenerative periodontal therapy^24^ photodynamic therapy in periodontitis^25^ and overall periodontal therapy publications in specific journals^26^ These analyses can improve the understanding of research activity in the topic explored, offer insights into the field’s evolution, identify the most productive authors, and provide information about promising avenues for further exploration and collaboration^27^ ^,^ ^28^ ^,^ ^29^. For instance, in the context of periodontal therapy, the conduct of randomized clinical trials (RCTs) is highly expected to directly impact patients’ treatment improvement. Still, in dentistry, a higher chance to conduct RCTs has been found among studies reporting some international collaboration^28^. However, no previous study explored the bibliometric trends and patterns of overall nonsurgical periodontal treatment studies, regardless of the journal. For these reasons, this bibliometric study aimed to map the scientific production of nonsurgical periodontitis treatment topics in terms of publication trends, patterns, and most productive researchers from 2001 to 2020. Moreover, this study investigated the factors associated with international collaboration and the chance to conduct RCTs in the nonsurgical periodontal therapy field.

## Material and methods

### Search strategy

A literature search was conducted in the Web of Science database ^27^ ^,^ ^30^ (September 27, 2021) using the following terms/query: periodontitis AND “non-surgical periodontal therapy” OR “non-surgical periodontal treatment” OR “nonsurgical periodontal therapy” OR “nonsurgical periodontal treatment” OR “scaling and root planning” OR “scaling and root planning” OR “subgingival instrumentation” OR “dental prophylaxis” OR toothbrush OR “interdental brush” OR “inter-dental cleaning” OR “dental floss” OR “biofilm control” OR “oral hygiene instruction” OR “mechanical plaque removal”. Filter was applied to the database to select papers published from the last two decades-2001 to 2020.

### Studies selection

The articles were selected by 4 independent calibrated examiners (V. L. A., M. H. R. B., D. V. A. P. D., and K. S.). The Cohen kappa was used to assess their agreement (k=0.90). Divergences between examiners were resolved by consensus. All articles were evaluated in terms of the following inclusion criteria: original research (clinical trials, *in vitro*, *in situ*, cross-sectional, longitudinal studies, animal models, case reports, case series, and systematic reviews), reviews, and the and the main goal of any outcome related to nonsurgical periodontitis treatment. Comments and letters to the editor were excluded. Studies evaluating nonsurgical treatments for gingivitis or other periodontal conditions were excluded. Studies using the general term “periodontal disease” without specifying the condition (i.e., periodontitis) were included. Studies that did not specify that was evaluating therapy for periodontal disease were excluded (i.e., overall biofilm reduction without specifying the condition/disease). No language restriction was applied. Initial screening considered the title and abstract to check whether the study met the inclusion and exclusion criteria. All divergencies were discussed by examiners for consensus. Then, all included studies were considered in the bibliometric analysis.

### Data extraction

For each article selected, the following information was collected: publication year and name of the journal, the title of publication, all authors and their respective countries (limited to the first 20 authors), number of authors, number of countries, number of institutions, number of citations on Web of Science, study design, continent of corresponding author, the main study focus/aim/topic, and funding reported. International collaboration was considered when at least two different countries were reported among authors' affiliations. The ten most productive authors' information was collected from the Scopus database (article number, citations, average of citations, and h-index).

### Normalization of data

The normalization of data included identifying variants and creating a standardization. Country of corresponding authors according to author affiliation. For authors with more than one affiliation, the affiliation described in the correspondence section was used(28). In cases where the country of the author or the corresponding author was not designated on the published paper, the Scopus database was used to investigate the affiliation and country. For the continent of the corresponding author, the categories were North America, Europe, Asia, South America, and Oceania/Africa. If the corresponding author had more than one affiliation on different continents, the continent of affiliation listed in the correspondence section was used^29^. The number of institutions was collected from each author's affiliation. If an author presented two or more affiliations in different institutions, only the first listed institution was considered^29^. The study design was categorized into (a) *in vitro*, (b) RCT, (c) N-RCT, (d) case report/case series, (e) retrospective study, (f) systematic review/meta-analysis, and (g) *in vivo* (*in situ*/animal). Research topics were categorized into (a) professional biofilm control, (b) patient biofilm control, (c) antibiotics (local or systemic), (d) other adjunctive methods (other than antibiotics), (e) lasers, and (f) others as detailed. When the study presented multiple topics, the most relevant was recorded according to the study objectives.

### Statistical analysis

GraphPad Prism 8.0 was used to generate the graphs. SPSS 21.0 (IBM Corporation) was used for statistical analysis. Descriptive analysis showing absolute values, percentages, averages, and standard deviation was used to summarize the data. Analytical statistical analysis considered two dependent variables: international collaboration (yes or no) and conduction of RCTs (yes or no). Both dependent variables were used to run all analyses separately. Then, multiple models identified the associations between international collaboration or conduction of RCTs with the following covariates: continent, study focus/aim, number of authors, number of institutions, and citation number. International collaboration was considered covariable when the conduct of RCTs was the dependent variable and the opposite. Logistic regression was used in the multiple models to identify the exploratory factors associated. Unadjusted and adjusted models were generated. A significance level of 5% was considered for adjusted models. Odds ratio values were estimated.

## Results

### Characteristics of the included studies and most productive authors

Among the 7,038 results using the search terms on Web of Science, 1,519 articles were included in the bibliometric analysis after screening and adopting inclusion and exclusion criteria. Among the included articles, 75.5% (1,147) had authors from a single country, and RCT was the most used design for nonsurgical periodontal treatment research (44.1%; n=669 studies). Professional biofilm control was the topic most studied (35.6%; n=541 studies). Concerning the geographic origins, Europe published the most significant number of articles (41.1%); n=625 studies (Table 1).

The ten most productive authors ranking according to the number of articles included resulted in the inclusion of 15 authors, since for some positions in the ranking ^8^ ^,^ ^9^ ^,^ ^10^, more than one author showed a similar number of articles included. Sculean A from the University of Bern (Switzerland) was the most productive author in nonsurgical periodontitis treatment from 2001 to 2020, showing 40 articles included in the study (Table 2), followed by Pradeep AR (Government Dental College and Research Institute, India) and Feres M (Guarulhos University, Brazil).

Journal of Periodontology was the most productive journal in this topic, publishing 19.7% of all articles included, followed by Journal of Clinical Periodontology and Journal of Periodontal Research (Table S1).

### International collaboration

**Figure 1:**
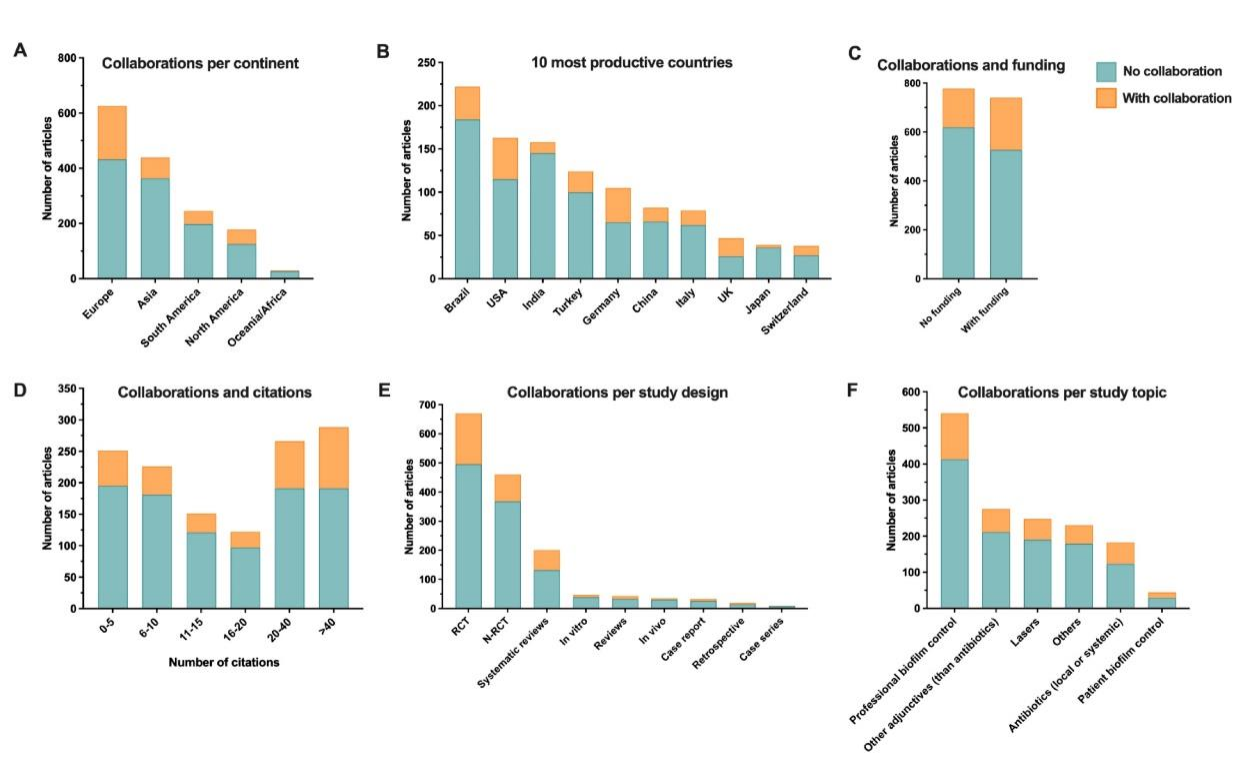
Distribution of articles according to the collaboration with other countries. A) Number of articles with and without international collaboration per continent; B) Number of publications with and without international collaboration of the 10 most productive countries in non-surgical periodontal therapy; C) Number of articles with and without international collaboration according to the funding status; D) Number of articles with and without international collaboration according to the number of citations; E) Number of articles with and without international collaboration per study design; F) Number of articles with and without international collaboration per study topic.

Studies with international collaboration had an average of 2.28 (( 0.72) countries collaborating. Europe was the continent with the highest number of studies with collaboration (Figure 1A). Brazil was the most productive country on periodontitis nonsurgical treatment from 2001 to 2020, but also showed the highest rate of no international collaboration among the 10 most productive countries (Figure 1B). Studies with some funding showed a slight increase for international collaboration (Figure 1C). Citations number (>40) increased with international collaboration (Figure 1D), and RCTs showed the highest number of studies with collaboration (Figure 1E). Considering the topic of the study, professional biofilm control showed a higher proportion of articles with some international collaboration (Figure 1F).

**Figure 2:**
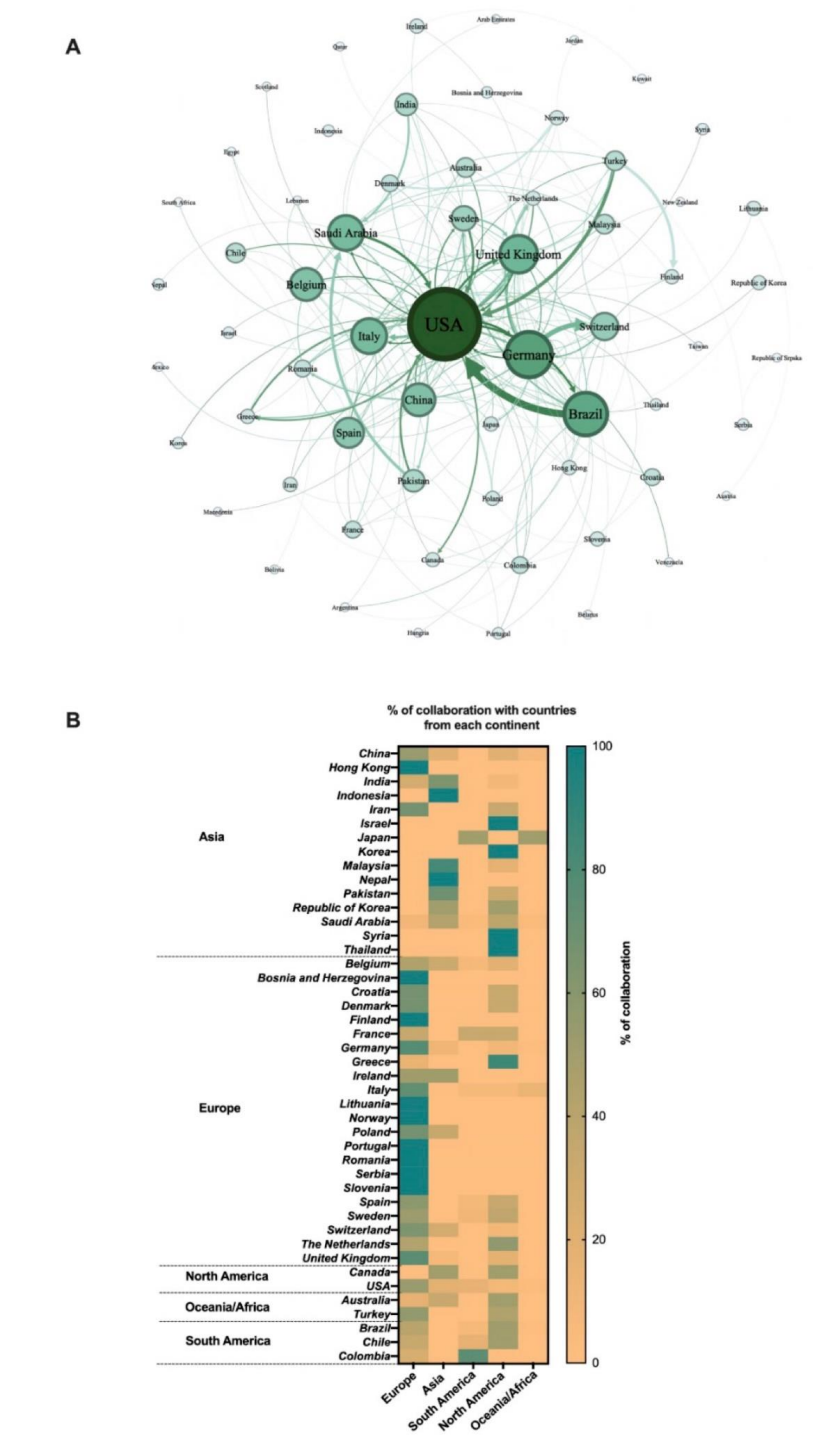
International collaboration in non-surgical periodontal therapy articles. A) Collaboration network among countries in articles related to non-surgical periodontal therapy (Note: As color and bubble size intensifies, the number of collaborations (articles) increases; as line thickens, the number of collaborations (articles) among countries increases). B) Countries that established international collaborations and % of collaborations with countries of each continent.

The United States of America (USA) was the country that collaborated more with other countries, mainly collaborating with authors from Brazil (Figure 2A). Although Europe was the continent with the most number of studies and collaborations, they collaborated intra-continent, except for Greece, which showed a high collaboration proportion with North America (Figure 2B).

For logistic regression analysis, unadjusted and adjusted models showed that international collaboration was associated (p<0.05) with the continent and number of institutions (Table 3). Asia (p<0.001), South America (p=0.004), and Oceania/Africa (p=0.016) showed lower chances of international collaboration compared to Europe. An increasing number of institutions in the articles were more likely to establish international collaborations (p<0.001).

**Table 1:** Descriptive analysis of included studies regarding periodontitis nonsurgical treatment from 2001 to 2020. N = 1,519.

*Variables*	*n*	*%*
*International collaboration*		
No	1,147	75.5
Yes	372	24.5
*Study design*		
In vitro	47	3.1
RCT	669	44.1
N-RCT	460	30.3
Case report/Case series	42	2.8
Retrospective study	20	1.3
Systematic review/Meta-analysis	244	16.1
In vivo (in situ, animal)	35	2.3
*Study design*		
No RCT	848	55.9
RCT	669	44.1
*Continent*		
Europe	625	41.1
North America	179	11.8
Asia	439	28.9
South America	246	16.2
Oceania/Africa	30	2.0
*Study focus/aim*		
Antibiotics	182	12.0
Professional biofilm control	541	35.6
Laser	247	16.3
Other adjunctives (than antibiotics)	275	18.1
Patient biofilm control	44	15.1
Others	230	2.9
		
	*Average*	*SD*
*Number of authors*	5.60	2.55
*Number of countries*	1.32	0.67
*Number of institutions*	2.14	1.47
*Citations*	29.79	42.44

### Conduction of RCTs studies

**Figure 3:**
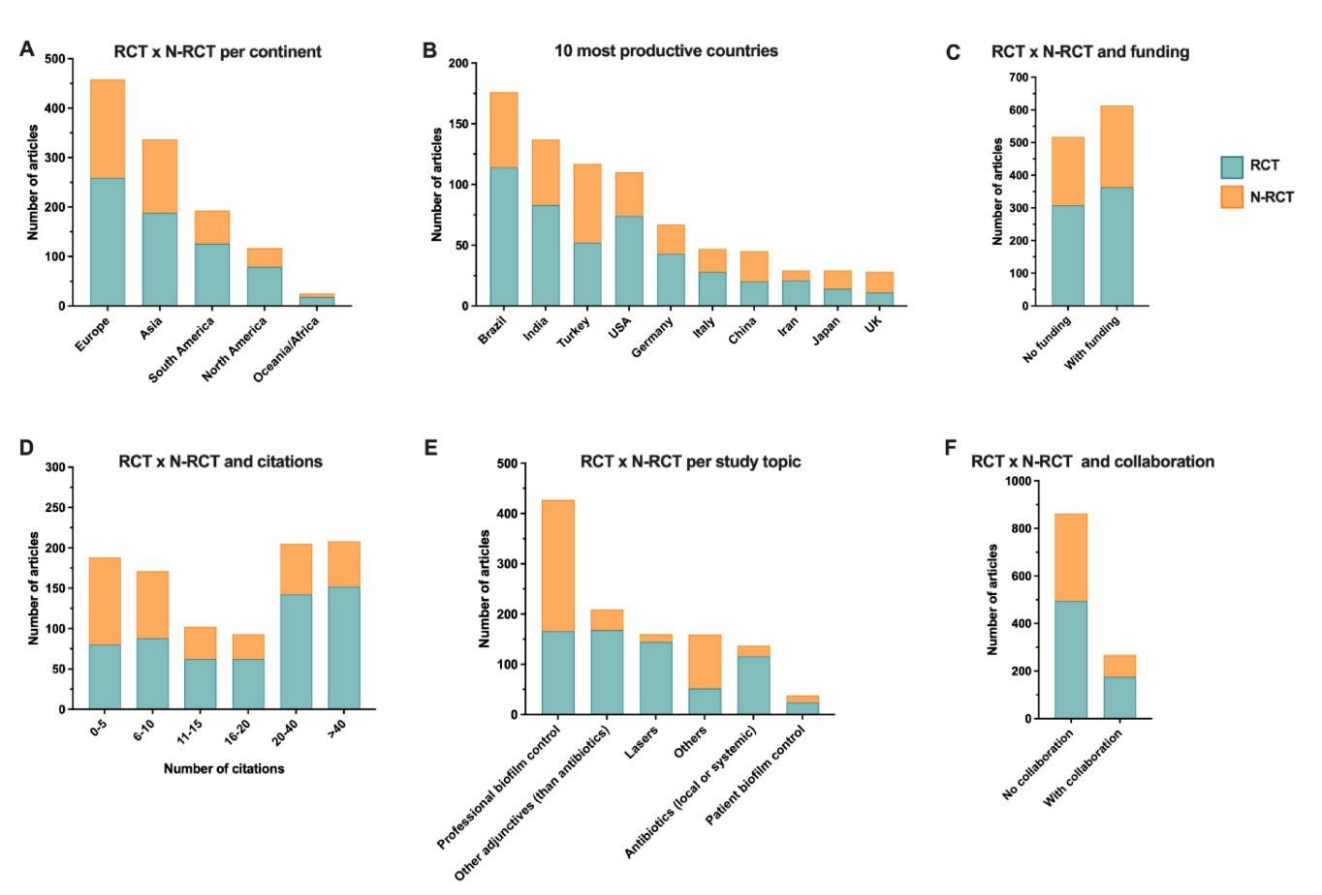
Distribution of RCT and N-RCT studies. A) Number of RCT and N-RCT studies per continent; B) Number of RCT and N-RCT studies of the 10 most productive countries in non-surgical periodontal therapy; C) Number of RCT and N-RCT studies according to the funding status; D) Number of RCT and N-RCT studies according to the number of citations; E) Number of RCT and N-RCT studies per study design; F) Number of RCT and N-RCT studies per study topic.

RCT was the most used study design, mainly in Europe and Asia (Figure 3A). Among the ten most productive countries, Brazil showed the highest number of RCTs (Figure 3B). Articles that reported some funding showed a slight increase for the RCTs design (Figure 3C). RCTs also showed a higher proportion of citations (Figure 3D), and the main topics of RCTs were professional biofilm control, other adjunctives (than antibiotics), and lasers (Figure 3E). Most of the RCTs included showed no international collaboration (Figure 3F).

Adjusted regression logistic showed that studies from North America were more likely to be RCTs than studies from Europe (p=0.050) (Table 5). Moreover, studies focusing on professional biofilm control (p<0.001) and other topics (p<0.001) were less likely to be evaluated by RCTs compared to antibiotics. Studies using RCT design were more likely to be conducted in articles with an increasing number of authors (p<0.001) (Table S2).

**Table 2:** Ranking of the ten most productive authors according to the number of included articles in the study.

	Author	Total of articles	Total of articles on Scopus	Institution	Country	Total of citations (included articles)	The average of citations included articles
1	A Sculean	40	481	University of Bern	Switzerland	1664	41,60
2	A R Pradeep	39	179	Government Dental College and Research Institute	India	988	25,33
3	M Feres	35	187	Guarulhos University	Brazil	1423	40,66
4	L C Figueiredo	30	126	Guarulhos University	Brazil	1207	40,23
5	M Faveri	26	102	Guarulhos University	Brazil	1100	42,31
6	F Javed	22	245	University of Rochester	USA	624	28,36
7	P M Duarte	21	135	Guarulhos University	Brazil	766	36,48
8	Z Akram	19	84	The University of Western Australia	Australia	608	32,00
	H F R Jentsch	19	72	University Hospital of Leipzig	Germany	267	14,05
	S S Socransky	19	335	The Forsyth Institute	USA	1121	59,00
9	F Vohra	16	262	King Saud University	Saudi Arabia	382	23,88
	C M Pannuti	16	139	University of Sao Paulo	Brazil	417	26,06
10	G Atilla	15	108	Ege University	Turkey	573	38,20
	L H Theodoro	15	88	São Paulo State University	Brazil	373	24,87
	M Z Casati	15	239	University of Campinas	Brazil	415	27,67

Notes: The total of articles represents the total of articles of each author included in the present bibliometric study; the total of articles on Scopus was collected from 23/June to 04/July/2022; the institution and the country were collected from the affiliation in the last article published by each author included in this study; the average of citations of the included studies was calculated considering the total of citations of the included studies from each author divided by the total of studies included.

**Table 3:** Results from multiple logistic regression showing unadjusted and adjusted (significance level 5%) models for factors associated with international collaboration.

	International collaboration		Unadjusted analysis		Adjusted analysis	
Variables	No (%)	Yes (%)	OR	p-value	OR	p-value
*Study design*						
In vitro	3.4	2.2	1			
RCT	43.2	46.8	1.06	0.893		
N-RCT	32.1	24.7	0.82	0.667		
Case report/Case series	3.0	2.2	0.58	0.392		
Retrospective study	1.2	1.6	1.33	0.670		
Systematic review/Meta-analysis	14.4	21.2	1.49	0.375		
In vivo (in situ, animal)	2.6	1.3	0.47	0.278		
*Continent*						
Europe	37.7	51.9	1		1	
North America	11.1	14.0	0.61	0.042	0.74	0.142
Asia	31.6	20.4	0.46	<0.001	0.47	*<0.001*
South America	17.3	12.9	0.56	0.009	0.57	*0.004*
Oceania/Africa	2.4	0.8	0.15	0.025	0.19	*0.016*
*Study focus/aim*						
Antibiotics	10.7	15.9	1			
Professional biofilm control	36.1	34.1	0.81	0.391		
Laser	16.6	15.3	0.65	0.118		
Other adjunctives (than antibiotics)	18.4	17.2	0.80	0.410		
Patient biofilm control	2.5	4.0	0.98	0.965		
Others	15.7	13.4	0.72	0.248		
*Number of authors*	5.46	6.02	0.94	0.118		
*Number of institutions*	1.86	2.98	1.80	<0.001	1.72	*<0.001*
*Citations*	28.4	33.73	1.00	0.256		

OR - odds ratio.

## Discussion

Although the management of a multifactorial condition such as periodontitis may require a combination of therapeutic approaches, it is expected to be the least invasive and most cost-effective protocol to deal with such a condition^17^ ^,^ ^31^. In this regard, nonsurgical therapies have been recommended as the second step in periodontal therapy, following patient-initiated behavioral changes aimed at enhancing oral hygiene and biofilm control to reduce subgingival biofilms^18^. Thus, evidence-based periodontology is expected to be a useful tool to support decision-making by integrating the current evidence into clinical practice^32^. In this sense, our findings may be helpful for researchers and clinicians to improve scientific production on this topic, pave ways for further studies, guide further research efforts, foster collaboration, and improve the dissemination of scientific information to enhance treatment outcomes. Interestingly, from 2001 to 2020, most of the articles focusing on nonsurgical periodontitis treatment were RCTs, which may aid in applying scientific information directly to clinical practice. However, almost half of the articles published are from European countries. Moreover, professional biofilm control was the main topic investigated, suggesting that researchers are still focusing on improving mechanical biofilm removal protocols to enhance clinical findings. Regression logistics showed that international collaboration in the included studies had occurred mainly among European countries and for studies with an increasing number of institutions; for the chance to conduct RCTs, it was higher for studies from North America and with an increased number of authors in the same article. Furthermore, the RCTs conduction chance was lower for studies focusing on professional biofilm control and other topics than antibiotics.

Among the most productive authors in the top 10 rankings, researchers from Europe, Asia, Oceania, and North and South America were included, highlighting a decentralization of leadership in this global topic. Since some authors showed a similar number of articles, the top 10 rankings had a total of 15 authors, with more than one author sharing the 8-10 positions. Anton Sculean, the most productive author in nonsurgical periodontitis treatment from 2001 to 2020, produced 2.6% of all articles included. Although the USA was the second most productive country and the main one collaborating with other countries, it is notable that only two researchers were included in the ranking. This may be explained by the lower success rate of funding from the National Institutes of Health (NIH) for less-prestigious institutions, as well as disparities in funding distribution among different states^33^. Moreover, NIH spending on projects focusing on oral disorders increased only 3% from 2008 to 2019^34^. Among the 15 authors included in the top 10 rankings, 7 are affiliated with institutions in Brazil. However, it should be noted that 4 of these authors are from the same university, Guarulhos University, and they shared the same publications related to nonsurgical periodontitis treatment topics.

A higher proportion of studies with international collaboration is expected in the biomedical field ^28^ ^,^ ^35^. 24.5% of included studies had some international collaboration, similar to that found for Implantology28. Interestingly, RCTs were the study design with a higher proportion of international collaboration, which may suggest the conduction of multicenter studies that can include more heterogeneous patients, improving treatment application in clinical practice^36^. These collaborations can directly affect the scientific quality, improving complexity, technologies applied, and even journal choice^28^. Furthermore, it is important to encourage and expand the connections between high-income and upper-middle and lower-middle-income countries to provide access to high-quality infrastructure and technologies^29^. However, the findings showed that authors from European countries predominantly collaborate within their own region. In contrast, the USA showed a broad range of collaborations, mainly establishing connections with authors from different countries in the nonsurgical periodontitis treatment field. In fact, countries from Asia, South America, and Oceania/Asia showed a lower likelihood of engaging in international collaboration than Europe. Nonetheless, as expected, the increasing number of institutions in the article showed a higher chance of international collaboration since more institutions can show more authors from different regions. Hence, for further studies considering authors from different institutions, it is anticipated that institutions from different countries will increase. This is because studies with international collaboration have demonstrated a higher citation number and have received funding, especially for RCTs.

RCTs are considered the gold standard on levels of evidence in healthcare settings since they provide essential information for developing new protocols, biological products, medical devices, and other innovations that can be directly implemented in clinical practice^37^ ^,^ ^38^. In the periodontology field, the clinical application of RCTs can be complex because of the heterogeneity and subjectivity of the outcome^38^. As expected, Europe showed a higher number of RCTs conducted, which may be explained by the higher production, expertise of researchers, funding available, and international collaboration. The RCTs evaluated topics related to biofilm removal and control, the main etiologic factor triggering periodontitis. Interestingly, North America exhibited a higher chance of conducting RCTs on nonsurgical periodontitis treatment topics. This can be attributed to the higher number of collaborations with other countries, which may result in the conduct of multicenter studies. However, this study design may require more resources, and previous evidence has shown that the USA received a higher level of resources, mainly among doctoral universities, compared to Europe^39^. Additionally, the lower chance of RCTs designed to evaluate professional biofilm control and other topics, compared to antibiotics, may be explained by the current use of systemic antibiotics to treat periodontitis, whose effectiveness can be evaluated only by RCTs^40^.

These findings can provide substantial information to guide further research on nonsurgical periodontitis treatment to improve therapeutic protocols and, consequently, patient care and disease burden. The gaps and trends identified here can influence researchers to align their work on priority areas and enhance technologies and applications; from the clinician's side, the most up-to-date treatment protocols developed by further studies can directly improve clinical practice and success rates, and policymakers can allocate adequate resources in the implementation or improvement of policies focusing on scientific development and health systems. Limitations of this study included that a specific period was considered (2001-2020), and this information needs to be constantly updated. Non-surgical periodontitis treatment remains a topic widely explored in the literature, focusing on the enhancement of therapeutic protocols and the understanding of disease outcomes. Approximately 600 papers have been found on the Web of Science per year using our search terms, indicating high scientific production. Therefore, the findings and trends presented here need to be constantly updated, especially regarding the new technologies and techniques used in recent studies, such as molecular analysis (i.e., RNA sequencing, proteomics). Moreover, some uncontrollable factors related to countries, such as population number, funding distribution, number of institutions, and number of doctoral degrees, were not considered. Notably, some information was considered based on the corresponding author, which may not represent all authors. Furthermore, other factors related to scientific production can also be associated with the outcomes and need to be considered by further studies, such as other databases and type of funding.

Based on the findings, this study concludes that from 2001 to 2020, the nonsurgical periodontitis treatment field has conducted mainly RCTs and focused on professional biofilm control. International collaboration was higher for studies with more institutions participating in the conduct and for studies conducted in Europe, but European countries have collaborated mainly intra-continent. The USA evidenced the highest level of international connections, mainly with Brazil, the most productive country in this topic. Moreover, RCTs have been conducted more by North American countries and by studies focusing on antibiotics. These findings highlight that scientific production on nonsurgical periodontitis treatment needs to be improved in terms of collaborations among research groups worldwide, mainly between high-income and upper-middle and lower-middle-income countries. Such collaboration can lead to the production of additional RCTs that can be applied in clinical practice. Furthermore, researchers should also prioritize exploring other relevant topics, including different adjuvant agents and integrating new technologies into periodontal therapy.
